# Single-center study of Lynch syndrome screening in colorectal polyps

**DOI:** 10.1186/s13053-019-0108-6

**Published:** 2019-03-12

**Authors:** FangChao Zhu, Da Pan, Hui Zhang, Qiong Ye, PeiSong Xu, Jie Pan

**Affiliations:** 1 Department of gastroenterology, WenZhou Central Hospital, WenZhou, ZheJiang Province China; 2 Department of Pathology, WenZhou Central Hospital, WenZhou, ZheJiang Province China; 3 HangZhou Zhiyuan Medical Laboratory Co., Ltd, WenZhou, ZheJiang Province China

**Keywords:** Lynch syndrome, Mismatch repair deficiency, Colorectal polyps, Immunohistochemistry, Genetic testing, Screening

## Abstract

**Background:**

Lynch syndrome is the most common hereditary colorectal cancer syndrome, and adenoma is one of the important premalignant lesions to colorectal cancer in Lynch syndrome. The first objective of this study was to calculate the detection rate of Lynch syndrome in colorectal polyps by using mismatch repair immunohistochemistry as the initial screening strategy. The second objective of this study was to optimize screening strategies for adenoma associated with Lynch syndrome by integrating polyp and/or patient characteristics such as polyp size, location, dysplasia, age of onset and/or family history of cancer.

**Methods:**

From June 2014 to May 2016, immunohistochemistry was performed for mismatch repair proteins (*MLH1, MSH2, MSH6,* and *PMS2*) using endoscopically resected specimens obtained from newly diagnosed colorectal adenomas. Gene analysis was performed in patients with missing expression of mismatched repair protein.

**Results:**

Five hundred and ten patients underwent colorectal polyp resection, with a total of 718 polyps. Five hundred and eight resected adenomas underwent mismatch repair protein immunohistochemical testing. Loss of mismatch repair protein expression was observed in six adenomas, accounting for 1.18% of all adenomas. Five patients then underwent genetic tests to identify two pathogenic mutations from different individuals, while another patient was suspected to have a pathogenic mutation. Three patients were younger than 50 years old. Two patients had advanced histology (high-grade dysplasia and malignant components) and one patient had a family history of cancer.

**Conclusion:**

Immunohistochemical detection of colorectal polyp mismatch repair protein as Lynch syndrome screening efficiency is low. Effective screening strategies may be improved by optimizing patient/polyp selection, such as focusing on young adenoma patients with a family history of cancer, or patients who present with high-risk features (large size, villous, high-grade dysplasia and malignant components).

## Introduction

Lynch syndrome (LS) is the most common disease that causes hereditary colorectal cancer syndrome, accounting for approximately 2–5% of all colorectal cancers [1], caused by autosomal dominant inheritance of a germline mutation in DNA mismatch repair (MMR) genes (*MLH1, MSH2, MSH6,*and *PMS2*) or *EPCAM* gene deletion. Immunohistochemistry (IHC) examining loss of expression (LOE) of one or more MMR proteins can be performed on adenomas as a screening test for LS, and subsequent genetic testing based on MMR IHC can ultimately confirm an LS diagnosis.

Colorectal adenoma is one of the most important precancerous lesions of colorectal cancer in LS. Compared with sporadic colorectal cancer, LS related adenoma can easily develop into invasive malignant tumor and accelerate canceration [2]. Adenomas in MMR mutation carriers have rates of MMR IHC deficiencies from 50 to 80% [3–5]. There seems to be a possibility of MMR IHC as a screening test for LS for premalignant polyps. However, some studies suggest the rate of LOE in MMR proteins or microsatellite instability (MSI) in sporadic adenomatous polyps is low (< 2%) [6–8]. Loukola et al. reported the frequency of MSI in adenomas was 1.6% (6/378); and in an unselected Finnish population, five out of six patients (83%) exhibited a germline MMR gene mutation [6]. In a recent study of 57 patients, the incidence of abnormal MMR IHC was 5.3%, but most of these patients had a family history of cancer or polyps [9]. The incidence of MMR IHC abnormalities in young patients was also low [7, 10]. The study on the LOE of MMR protein in Chinese polyps is not clear, so we have carried out MMR IHC, and genetic analysis of patients with LOE of MMR protein.

## Materials and methods

### Patient selection

Between June 2014 and May 2016, Five hundred and ten newly diagnosed colorectal polyps underwent endoscopic polypectomy. The clinicopathological data and family history of cancer were obtained from electronic medical records.

### IHC for mismatch repair proteins

If a patient presented more than one adenoma, we chose the more advanced adenoma for MMR IHC testing. Five hundred and eight adenomas from 508 patients were analyzed by MMR IHC; however, one patient had an inflammatory polyp and one a hyperplastic polyp. IHC was performed on four MMR proteins, *MLH1, MSH2, MSH6,* and *PMS2*, using a Staining Automat (Benchmark), according to the manufacturer’s protocol. We obtained 4-μm-thick formalin fixed paraffin sections. Primary antibodies used to detect MMR proteins are included anti-hMLH1 antibody (clone ES05; ZSGB-Bio Ltd., Beijing, China; 1:50), anti-hMSH2 antibody (clone RED2; ZSGB-Bio Ltd., Beijing, China; 1:100), anti-hMSH6 antibody (clone UMAB258; ZSGB-Bio Ltd., Beijing, China;1:100), and anti-hPMS2 antibody (clone M0R4G; ZSGB-Bio Ltd., Beijing, China; 1: 40). Nuclear staining of non-tumor cells such as normal colonic epithelial cells, lymphoma cells, or stromal cells served as an internal positive control. We judged that there was no nuclear staining at all in tumor cells and suggested that MMR was deficient. Staining results were assessed by consensus by two independent pathologists (Qiong Ye and Yi Jiang).

### DNA extraction and sequencing

Patients with a defect mismatch repair (dMMR) polyp were referred to as genetic counseling, and informed consent was signed for gene detection. DNA was extracted from leukocytes using the QIAamp DNA Mini kit (Qiagen GmbH, Hilden, German). Germline testing was targeted towards the MMR IHC pattern. Specific primer synthesis and Sanger sequencing were performed by HangZhou Zhiyuan Medical Laboratory Co., Ltd. Amplified DNA fragments included coding exons, flanking intron regions, and promoter regions. Four patients underwent Sanger sequencing by using an ABI 3730XL fully automated DNA sequencer (Thermo Fisher Scientific Inc.). Only one patient underwent next-generation sequencing (NGS) by Beijing Deyidongfang Translational Medicine Research Center Co., Ltd. InSiGHT, and at the same time, ClinVar databases were scanned to determine the significance of mutation.

### Statistical analysis

Data were expressed as medians and ranges. SPSS19.0 software was used for statistical analysis.

## Results

### Clinicopathologic characteristics

Five hundred and ten patients underwent colorectal polyp resection between June 2014 and May 2016. There were 375 men and 135women. The median age of the patients was 60 years (range: 28–89). Seven hundred eighteen polyps were resected, including 343 patients with one polyp, and 167 patients with more than one polyp. The pathological features of the patients included in this study are summarized in Table 1. The histological types were as follows: tubular adenoma (578 cases), villous adenoma (including tubulovillous) (107 cases), serrated adenoma (6 cases), inflammatory polyp (15 cases) and hyperplastic polyp (12 cases). Five adenomas presented malignant components. The site of the polyps was distributed as follows: left colon (proximal to splenic flexure), 496 cases; right colon (splenic flexure and distal to the splenic flexure), 222 cases.

**Table 1:** Pathological characteristics of colorectal polyps

Category	Number
Histologic classification	
Adenoma	691
Tubular adenoma	578
Serrated adenoma	6
Villous adenoma (including tubulovillous)	107
Inflammatory polyp	15
Hyperplastic polyp	12
High-grade dysplasia	44
Malignant polyp	5
Location	
Left colon (proximal to splenic flexure)	496
Right colon (splenic flexure and distal to the splenic flexure)	222

### IHC for MMR proteins

Clinicopathologic characteristics of six patients with LOE of MMR proteins are summarized in Table 2. LOE of MMR proteins was observed in 6 adenomas accounting for 1.18% of the patient population.

**Table 2:** Detailed data of the six patients with LOE of MMR proteins

Nr	Sex	Age(years)	Site	Size	Pathology	Pattern of MMR protein loss	Mutation	Amino acid change	Clinical significance of mutation	Family history of cancer
1.	F	45	rectum	1 .2cm	tubulovillous adenoma with high-grade dysplasia	*MSH2MSH6*	*MSH6* deletion of exon7	unknown	possibly pathogenicity	No tumors in first- or second-degree
2.	F	65	sigmoid colon	0.6 cm	tubular adenoma with low-grade dysplasia	*MSH2*	negative	NA	NA	No tumors in first- or second-degree
3.	M	58	transverse colon	3 cm	tubular adenoma with high –grade dysplasia	*MLH1PMS2*	Not detected	NA	NA	unknown
4.	M	48	rectum	1 .5cm	tubular adenoma,malignant	*MSH2*	c.181C > T (NM_000251.2)	p.Gln61Ter	pathological	No tumors in first- or second-degree
5.	M	48	transverse colon	0 .8cm	tubular adenoma with low -grade dysplasia	*MSH2MSH6*	*MSH2*c.842C > G (NM_000251.2)	p.Ser281Ter	pathological	Father died of colon cancer at the age of 58;two uncles with colon cancer
6.	M	39	ascending colon	3 .5cm	tubular adenoma with low–grade dysplasia	*MSH2*	c.1168C > T (NM_000251.2)	p.Leu390Phe	benign variant	Four uncles with colorectal cancer

### Identification of LS

In 6 patients with LOE of MMR proteins, one patient refused to be tested for gene, and the other 5 patients were tested. Table 2 summarizes the results of genetic testing.

Three patients who underwent Sanger sequencing had three mutations in *MSH2*, two pathogenic mutations (c.181C > T,c.842 C > G), and one benign mutation. A patient who received Sanger bidirectional sequencing showed a deletion of exon 7 in MSH6, but he declined further multiplex dependent probe amplification testing. So we speculated that he had a pathogenic mutation. Another patient received NGS did not have mutation in MSH2.

## Discussion

In the past, the initial diagnosis of Lynch syndrome was based on family history of cancer, according to the Amsterdam II standard or the Bethesda revision criteria. The final diagnosis was established by identification of a germline heterozygous pathogenic mutation in *MLH1, MSH2,* or *MSH6*, or an *EPCAM* deletion upon molecular genetic testing. However, modern Chinese families tend to be small, which may lead to incomplete penetrance of MMR mutations that make it difficult to recognize LS. Studies have shown that these criterias are less sensitive and/or specific [11, 12]. Currently, guidelines and studies strongly recommend LS screening for all newly diagnosed colorectal cancer patients using MMR,IHC and/or MSI tests [13–15]. Early diagnosis of LS patients is important; Heikki and Jarvinen et al. have shown that 15-year follow-up of colonoscopy in LS families effectively reduced mortality by 65% [16].

Adenoma is the primary pre-malignant lesion of Lynch syndrome. In one study concerning MMR gene germline mutation carriers, MSI was detected at 44% in low-grade epithelial neoplasia, and 100% in high-grade dysplasiaThe frequency of MSI in LS-related adenoma was 41–80% [17–22], and the risk of MSI was 3.6-fold higher than that of non-carriers. By the age of 60, the cumulative rate of adenoma in the mutation carrier was 70% [19]. MSI is an early molecular event in the LS-related adenoma. In one study concerning MMR gene germline mutation carriers, MSI was detected at 44% in low-grade epithelial neoplasia, and 100% in high-grade dysplasia, suggesting that the MSI may not be the first, but it is possible to accelerate the progression of the adenoma to the invasive cancer [17]. To better understand colorectal carcinogenesis in LS, Aysel Ahadova et al. demonstrated that LS colorectal cancers can develop through three pathways. Some colorectal cancers in LS can in fact grow out from MMR-proficient adenomas after secondary inactivation of the MMR system; however, a larger portion of cancers appears to develop from mismatch repair deficient crypt focus (MMR-DCF), either through an adenomatous phase or as nonpolypous lesions with immediate invasive growth [23]. Hence, dMMR is a key characteristic of LS-related colorectal adenoma.

Because, adenomas with LOE of MMR proteins evolve to adenocarcinoma quickly, we need an early screening strategy to identify these adenomas. However, there is no clear screening strategy for LS in colorectal adenomas. A study of adenoma characteristics of MMR germline mutation carriers found that these adenomas are more common in the right colon, are generally ≥7 mm, and consist of high-grade intraepithelial neoplasia [22]. Detection of LS by only performing MMR IHC and/or MSI testing of adenomas in young patients may not be the best strategy. In patients with colorectal adenoma < 40 years old, MMR IHC and MSI showed no abnormal results [7]. In 208 patients < 40 years-old with colorectal adenoma, only one patient (0.4%) exhibited LOE of MMR (MLH1 PSM2) proteins [10]. In our study, we found that the LOE of MMR proteins was mainly distributed in MSH-2 and MSH-6 in 6 adenomas by MMR IHC detection in 508 inpatients with adenomas. There was no difference in the distribution of the left and right hemicolon among the six adenomas. The average age was 50 years, the mean size of adenoma was 1 .7cm. Two adenomas showed high-grade dysplasia, and one adenoma showed malignant component. The remaining two adenomas showed no progression, but came from young patients with a family history of cancer and met the Bethesda revised criteria. Age of onset and the size of adenomas may be key factors in predicting LOE of MMR proteins, but a good strategy for detection of LS also needs to include advanced histology (high-grade dysplasia and malignant components) and a family history of colorectal cancer.

Ultimately, five patients underwent genetic testing. Two patients with LOE of the *MSH2* protein had pathogenic mutations,c.181C > T and c.842C > G. Amino acid changes were p.Gln61Ter and p.Ser281Ter, which caused termination of amino acid translation. One case of MSH 6 exon 7 deletion mutation. The patient refused to further verify the multiple -dependent probe amplification test. Three cases were younger than 50 years old, 2 cases had advanced histology (high-grade dysplasia and malignant components), and 1 case had a family history of cancer.

Our study has limitations. First, MMR-deficient nonpolypous is another important precursor in LS; so our screening for LS from adenomatous polyps indeed may have missed quite a few cases. Second, our immunohistochemical screening strategy may also missed a few cases; genetic testing of all adenomatous polyps is the most accurate strategy.

## Conclusion

Currently, the study contains the largest sample size, screening for Lynch syndrome by MMR IHC on colorectal polyps. The results suggest that when young adenoma patients have a family history of LS associated cancer, or young adenoma patients have high risk characteristics, such as large volume, high grade dysplasia and malignant components, it may be reasonable to evaluate LS.

## Abbreviations

dMMR: Deficient mismatch repair
IHC: Immunohistochemical
LOE: Loss of expression
LS: Lynch syndrome
MMR: Mismatch repair
MSI: Microsatellite instability
NGS: Next generation sequencing
